# Heart Failure with Preserved Ejection Fraction—a Concise Review

**DOI:** 10.1007/s11886-020-01349-3

**Published:** 2020-07-09

**Authors:** Daria M. Adamczak, Mary-Tiffany Oduah, Thomas Kiebalo, Sonia Nartowicz, Marcin Bęben, Mateusz Pochylski, Aleksandra Ciepłucha, Adrian Gwizdała, Maciej Lesiak, Ewa Straburzyńska-Migaj

**Affiliations:** 1 Ist Department of Cardiology, Poznan University of Medical Sciences, Dluga Street ½, 61-848 Poznan, Poland; 2 Center for Medical Education in English, Poznan University of Medical Sciences, Poznan, Poland; 3 Faculty of Medicine, Poznan University of Medical Sciences, Poznan, Poland

**Keywords:** HFpEF, Heart failure, Diastolic dysfunction, Heart failure with preserved ejection fraction, Preserved left ventricular function

## Abstract

**Purpose of Review:**

Heart failure with preserved ejection fraction (HFpEF) is a relatively new disease entity used in medical terminology; however, both the number of patients and its clinical significance are growing. HFpEF used to be seen as a mild condition; however, the symptoms and quality of life of the patients are comparable to those with reduced ejection fraction. The disease is much more complex than previously thought. In this article, information surrounding the etiology, diagnosis, prognosis, and possible therapeutic options of HFpEF are reviewed and summarized.

**Recent Findings:**

It has recently been proposed that heart failure (HF) is rather a heterogeneous syndrome with a spectrum of overlapping and distinct characteristics. HFpEF itself can be distilled into different phenotypes based on the underlying biology. The etiological factors of HFpEF are unclear; however, systemic low-grade inflammation and microvascular damage as a consequence of comorbidities associated with endothelial dysfunction, oxidative stress, myocardial remodeling, and fibrosis are considered to play a crucial role in the pathogenesis of a disease. The H_2_FPEF score and the HFpEF nomogram are recently validated highly sensitive tools employed for risk assessment of subclinical heart failure.

**Summary:**

Despite numerous studies, there is still no evidence-based pharmacotherapy for HFpEF and the mortality and morbidity associated with HFpEF remain high. A better understanding of the etiological factors, the impact of comorbidities, the phenotypes of the disease, and implementation of machine learning algorithms may play a key role in the development of future therapeutic strategies.

## Introduction

The spectrum of disorders involving myocardial dysfunction with typical signs and symptoms has since been referred to as heart failure (HF) [1, 2]. Echocardiographic parameters, i.e., ejection fraction (EF), have been used for subclassification of this complex clinical entity: heart failure with reduced EF (HFrEF; EF < 40%), mid-range EF (HFmrEF; EF 41–49%), and preserved EF (HFpEF; EF ≥ 50%) have all been recognized as different points on the continuum of heart failure disorders [3].

Heart failure has been increasingly recognized as an epidemic and various possible etiologies have now been identified. These include coronary artery disease, valvular heart disease, hypertension, cardiomyopathies, and adverse effects of drugs and toxins [3]. In developing and developed countries, heart failure incidence continues to rise, accounting for most cases of HF in the developed world [2, 3]. HFpEF was discovered by Dr. Luchi et al., who in 1982 described a group of patients with typical heart failure symptoms and associated preserved (≥ 50%) left ventricular ejection fraction (LVEF) [4]. Recently, HFpEF has been defined by the European Society of Cardiology (ESC) as preserved left ventricular EF (LVEF ≥ 50%), with evidence of diastolic dysfunction or structural heart disease, in the context of classic signs and symptoms of heart failure and elevated natriuretic peptides [3, 5].

The complex interplay between various factors involved in the etiopathogenesis and potentiation of heart failure has sparked a new drive for heart failure classification based on various (molecular and biochemical) parameters and biomarker profiles [6, 7•]. Indeed, the inter- and intra-observer reliability of LVEF has been noted to vary markedly, thus diminishing the clinical utility of LVEF for diagnostic and prognostic purposes [7•]. However, the terms HFrEF, HFmrEF, and HFpEF will be used in our descriptions for simplicity.

## Prevalence and Demographics

The prevalence of HF is estimated to be 1.1–5.5% in the general population [8]. It is a common cause of hospitalization. Those who are diagnosed with HFpEF represent about a third to one-half of the total number of HF patients [9–11]. Current data suggests that there is a shift in the type of heart failure patients are likely to be diagnosed with. Epidemiological data revealed that the prevalence of HFpEF relative to HFrEF is increasing at a rate of 1% per year, indicating that HFpEF is becoming the most common type of HF [8]. The highest rate of HFpEF is among the elderly; however, the younger subgroup of patients (< 65-year-old) accounts for 40% of all total cases [9, 12]. HFpEF affects more women than men, suggesting that gender may play a major role in disease evolution [13]. On the other hand, incidence rates are similar across all races and ethnicities [14]. Although patients with HFpEF have a lower risk of death than patients with HFrEF (HR 0.62, 95% CI 0.46–0.85), regardless of age, gender, or etiology of HF, absolute mortality is still high [15].

## Etiology

The etiology and pathophysiology of HFpEF are still being uncovered. Firstly, the etiological factors affecting HFpEF and HFrEF seem to be different [16]. The Framingham Heart Study suggests that the classification of HF be made depending on the underlying cause of the disease: coronary artery disease, valvular heart disease, hypertension, or other causes [16, 17]. Patients with HFpEF are more likely to have valvular heart disease, hypertension, and atrial fibrillation (*p* = 0.05, *p* < 0.001, and *p* < 0.001, respectively). On the contrary, patients with HFpEF are less likely to have a myocardial infarction or left bundle branch block (LBBB) (OR 0.21, 95% CI, 0.10–0.46, *p* < 0.001). Compared to patients with HFrEF, patients with HFpEF have significantly higher blood pressure (*p* = 0.04), lower resting heart rate, and lower levels of potassium in the plasma [16]. Many studies point out that patients with HFpEF are usually older women with hypertension [15, 16, 18, 19]. Indeed, arterial hypertension is one of the main factors leading to increased stiffness of blood vessels and increased afterload of LV [20].

Furthermore, comorbidities seem to play a pivotal role in the pathophysiology of HFpEF. The most common are obesity, diabetes, atrial fibrillation, metabolic syndrome, chronic obstructive pulmonary disease, sleep-disordered breathing, renal dysfunction, and anemia [7•, 21–27]. Aging seems to have a great impact as well [10, 28, 29].

Although the pathophysiology of HFpEF is yet to be understood, systemic low-grade inflammation, mediated through tumor necrosis factor (TNF) alpha and transforming growth factor (TGF) beta 1, was proposed as a cause of disease [18, 30]. However, the degree of diffuse myocardial fibrosis is not related to the severity of impairment of diastolic function in HFpEF [31]. Microvascular dysfunction induces systemic inflammation which is present before the clinical symptoms [18, 32]. It is mediated by microRNAs and the formation of different miRNA [18, 33]. Furthermore, the intrinsic cardiomyocyte phenotype is distinct in HFpEF and HFrEF. Research by Curl et al. indicates that hypertrophic heart rat (HHR) shows a significantly elevated calcium (Ca^2+^) operating level and increased L-type calcium channel current, which contrasts with the suppressed Ca^2+^ cycling state typical for HFrEF [34].

## Clinical Manifestations

The initial presenting symptoms of HFpEF may be included in the broad category of heart failure. Dyspnea is the most common manifesting symptom among them. Shortness of breath can manifest in various ways, whether it be upon exertion or at rest as in paroxysmal nocturnal dyspnea or orthopnea. Other non-specific symptoms such as fatigue are present. The typical heart failure symptoms such as ankle edema and jugular venous distention are often not present. Other possible presentations include decreased exercise tolerance, chest pain, or discomfort.

## Diagnosis

**Fig. 1:**
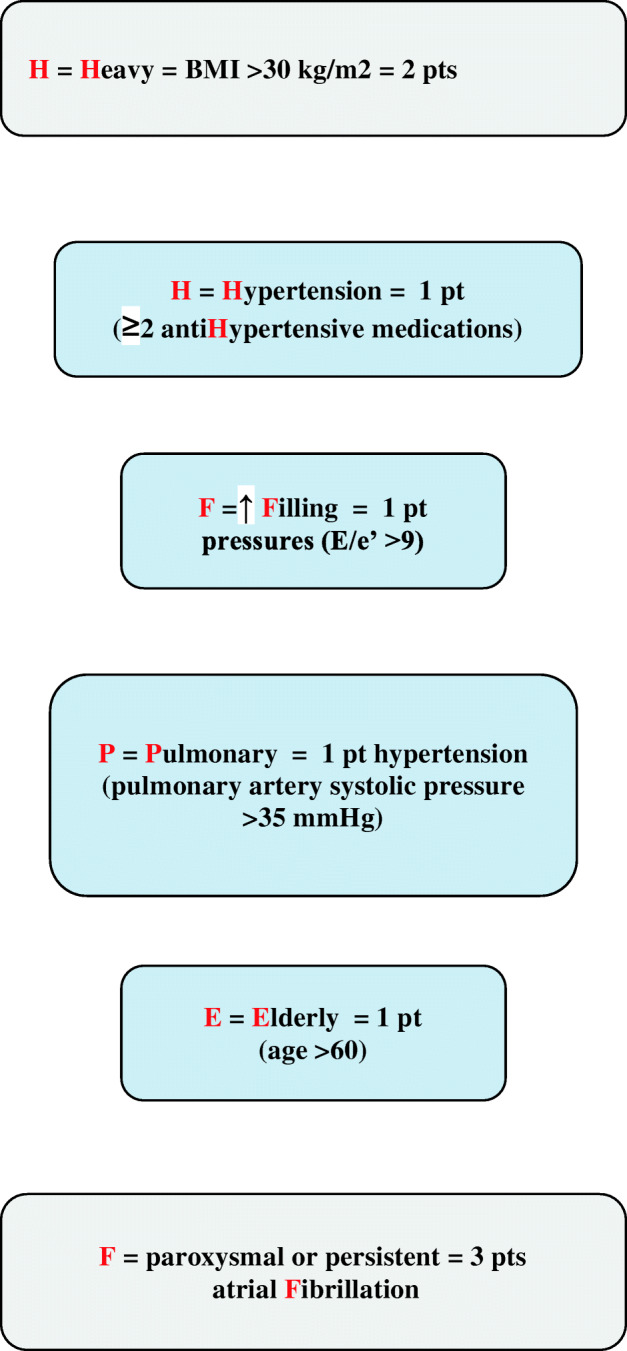
H_2_FPEF score used to determine the probability of HFpEF (figure created based on text from Paulus [35•])

The H_2_FPEF score and the HFpEF nomogram are recently validated highly sensitive tools employed for risk assessment of subclinical heart failure. These tools are based on clinical and echocardiographic parameters, including body mass index (BMI) > 30 kg/m^2^ (H); use of 2 or more antihypertensive medications (H); the presence of atrial fibrillation (F); pulmonary hypertension (pulmonary artery systolic pressure > 35 mmHg) (P); elderly with an age > 60 years (E); and elevated filling pressures (*E*/*e*′ > 9) (F). The H_2_FPEF score determines the probability of HFpEF by assigning a number for each item (Fig. 1) [35•]. Although formerly designated as *heart failure with diastolic dysfunction*, HFpEF may occur in the absence of signs of diastolic dysfunction, and as such evidence/presence of diastolic dysfunction is not required for the diagnosis [36].

As previously mentioned, the European Society of Cardiology (ESC) guidelines for the diagnosis of HFpEF include left ventricular EF (LVEF) ≥ 50%, evidence of either diastolic dysfunction or structural heart disease, signs and/or symptoms of heart failure, and elevated natriuretic peptides [3]. Given the complexity of HFpEF, various parameters including clinical (patient history and physical examination), biochemical (serum BNP level), hemodynamic, and radiographic data are utilized in reaching a diagnosis [37, 38]. Oftentimes exercise testing is required to confirm the diagnosis when signs of diastolic dysfunction occur only on exertion but not at rest. Nevertheless, the echocardiographic evaluation is crucial, and advanced techniques seem particularly promising. Shah et al. proposed recently that echocardiography could serve as a “digital biopsy” of the heart. Speckle-tracking echocardiography (STE) can be utilized to assess cardiomyocyte calcium homeostasis, excitation-contraction coupling, and the health of T-tubules before the onset of myocardial fibrosis [39, 40]. Furthermore, defining left atrial structure and function has recently gained importance in evaluation of LV diastolic dysfunction [41].

**Fig. 2:**
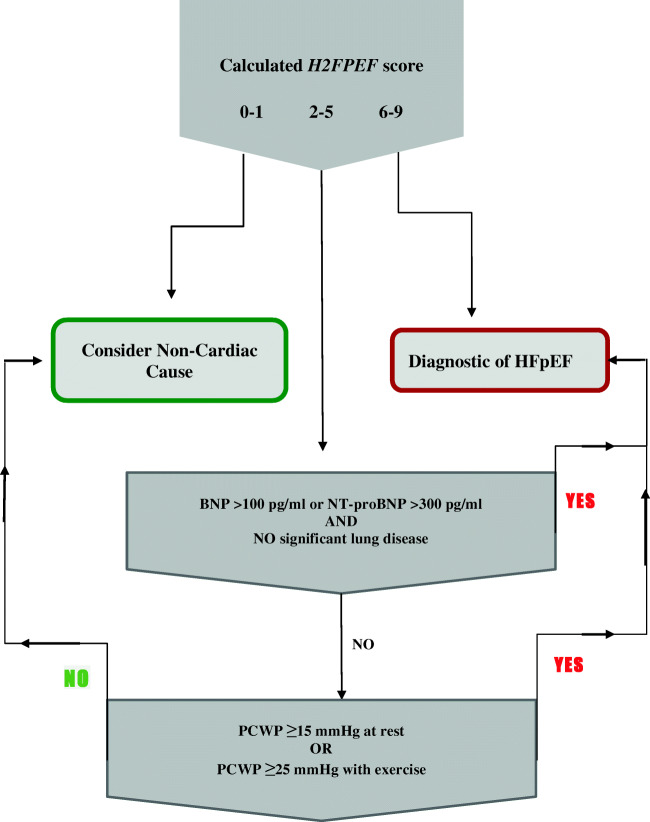
Diagnostic algorithm for HFpEF (figure created based on text from Huis in’t Veld et al. [38])

Since HFpEF may also share similar clinical characteristics with valvular heart disease, pericardial disease, and high-output HF [42], diagnostic algorithms are useful in making the diagnosis of HFpEF (Fig. 2).

## HFpEF Phenotypes

Phenotypic presentations of HFpEF may vary widely across patients and determine the choice of diagnostic tests and targeted management plan [39••, 43–46]. There are four clinically distinct phenotypes of HFpEF that have been recognized [47]:

- Aging phenotype
- Obesity phenotype
- Pulmonary hypertension (PH) phenotype
- Coronary artery disease (CAD) phenotype

Although this classification acknowledges the heterogeneity and need for individualized approach, the biological phenotypes seem to better describe the underlying pathomechanisms of HFpEF. Shah et al. proposed a classification created by the use of machine learning [39••, 45, 48]:

The three identified biological phenogroups are as follows:

1. Natriuretic peptide deficiency syndrome—younger subjects with moderate diastolic dysfunction and relatively normal BNP
2. Extreme cardiometabolic syndrome—obese, diabetic subjects with a high prevalence of obstructive sleep apnea
3. Right ventricle-cardio-abdomino-renal syndrome—older subjects with significant chronic kidney disease and cardiopulmonary comorbidities

Worse outcomes are observed in phenogroups 2 and 3.

It is generally thought that the heterogeneity in the clinical presentation of HFpEF may be explained by underlying comorbidities in individual subjects. Thus, phenogrouping enables risk stratification and the institution of better-targeted therapies as opposed to BNP-based stratification alone [45].

## Differential Diagnosis

Since symptoms of HFpEF are non-specific, diagnosis might be elusive. The majority of patients complain of exertional dyspnea, which is a common cause of hospital admission. As such, there are multiple differential diagnoses to consider, including pulmonary and cardiovascular causes, or vocal cord conditions [49]. Differential diagnosis to rule out other causes of dyspnea should be based on echocardiographic examination and tissue doppler imaging [50]. Overall, clinicians should pay attention to non-specific manifestations of HFpEF and diagnose sensibly based on imaging studies.

## Evaluation of Comorbidities

In recent years, a new paradigm of HFpEF has been suggested, implying that it is a very heterogeneous disease. It can be caused by comorbidities through systemic endothelial inflammation leading to structural and functional remodeling of the heart [51].

The most significant comorbidities are obesity, diabetes, metabolic syndrome, chronic obstructive pulmonary disease, sleep-disordered breathing, renal dysfunction, and anemia [7•, 21–27]. Excess visceral fat leads to increased levels of proinflammatory cytokines [52]. Hyperglycemia, hyperinsulinemia, and insulin resistance lead to mitochondrial and microvascular dysfunction, as well as autonomic neuropathy, which cause cardiac stiffness, hypertrophy, fibrosis, and eventually HF [53]. It is worth noting that proper diagnosis and an understanding of comorbidities can significantly contribute to improvement in HFpEF patients’ outcomes [27].

## HFpEF and Hypertension

Chronic maladaptive neurohumoral activation leading to sustained systemic arterial hypertension has been implicated in the course of HFpEF [54]. Studies have shown that systemic hypertension is a critical determinant of outcome in HFpEF as it plays a crucial role in the onset and maintenance of a proinflammatory state, arterial stiffness, ventricular hypertrophy, titin-dependent stiffness, and dysfunction [55–57]. In patients with HFpEF, control of hypertension can induce regression of myocardial mass and improve cardiac function and relaxation as well as clinical outcomes [58, 59]. Thus, if concomitant hypertensive disease exists, it is crucial to introduce medical therapy in order to achieve lower blood pressure targets and prevent the untoward complications of increased afterload [56, 60]. According to ALLHAT trial, HFpEF patients have a more favorable prognosis than HFrEF counterparts, even among high-risk hypertensive patients [61, 62].

## HFpEF and Amyloidosis

Two types of amyloid commonly infiltrate the myocardium—immunoglobulin light chain (AL or primary systemic) amyloid and transthyretin (TTR) amyloid. Transthyretin-related amyloidoses (ATTR) may be either hereditary (caused by autosomal dominant mutations in the TTR gene) or acquired (due to misaggregation of wild-type transthyretin). ATTR amyloidosis is an increasingly common cause of HFpEF and must be excluded in patients suspected of HF [63, 64]. The amyloid is deposited in the myocardium and/or peripheral nervous system [65]. The most common cardiac symptoms are dyspnea, angina, edema, and syncope [66]. Non-cardiac manifestations include peripheral neuropathy, characterized by symptoms of neuropathic pain, numbness, and loss of muscle strength in the lower extremities. Gastrointestinal symptoms such as diarrhea and weight loss result as a consequence of autonomic neuropathy or autonomic nerve dysfunction of unknown etiology [67, 68]. Other autonomic manifestations include erectile dysfunction, orthostatic hypotension, and neurogenic bladder [69]. In addition, symptoms such as lumbar spinal stenosis may appear [70, 71]. Distal biceps tendon spontaneous rupture is also common in patients with transthyretin cardiac amyloidosis [72]. Ando et al. have also reported vitreous body inclusions of the cotton wool type, which are pathognomonic for ATTR amyloidosis [69]. Carpal tunnel syndrome (CTS) is an early presenting sign of disease, preceding the onset of HF by up to 5–9 years [73]. The prevalence of ATTR amyloidosis among patients with CTS is 7–8%, compared to 4–5% in the general population [74, 75]. CTS manifests as pain and sensory disturbances in the lateral distribution of the hand, as well as hand weakness observed in cases of severe focal neuropathy [76]. Biopsy and histopathologic analysis used to be required to identify amyloidosis. Congo red or Direct Fast Scarlet 4BS staining binds to amyloid fibrils and characteristic apple-green birefringence under polarized light microscopy is noted. However, imaging techniques as well as genetic testing are becoming increasingly important [77–79]. Echocardiography and cardiac magnetic resonance may reveal features suggestive of amyloidosis, such as thickened LV wall, atrial septum and valves, small LV cavity size, biatrial enlargement, elevated RV systolic pressure, granular sparkling appearance of the myocardial wall, pericardial effusion, restrictive filling pattern, and reduced ventricular strain with relative apical sparing pattern. However, it is not sufficient for the diagnosis [80–83]. Nuclear imaging techniques employing technetium-99 (^99m^Tc) labeled diphosphonopropanodicarboxylic acid (^99m^Tc-DPD), pyrophosphate (^99m^Tc-PYP), or methylenediphosphonic acid (^99m^Tc-MDP), once used as a bone scintigraphy, provide a novel, non-invasive diagnostic approach with relatively high sensitivity (> 90%) and specificity (86%) [84, 85]. Intense uptake of ^99m^Tc-DPD in the myocardium with lower or absent uptake in the bones suggests ATTR amyloidosis. Positive bone scintigraphy in patients without monoclonal gammopathy characterizes 100% specificity [86]. It enables to establish the diagnosis without the need of histology [84].

## Treatment

There is no evidence that medications, which are known to be effective at alleviating symptom burden and reducing mortality in patients with HFrEF, are equally effective for patients with HFpEF. It may be due to the disparateness of the disease as well as multifactorial pathophysiology of the disease [87]. The number of available clinical trials on the treatment of HFpEF is finite. Currently, angiotensin-converting enzyme blockers (ACEIs), angiotensin receptor blockers (ARBs), calcium channel blockers (CCBs), and beta-blockers are given to these patients, although trials with perindopril, candesartan, irbesartan, and nebivolol did not show a clear advantage over placebo [88–93]. On the contrary, spironolactone may be effective in HFpEF treatment. The TOPCAT randomized double-blinded study had as its aim to determine what effect spironolactone would have on HFpEF in regard to mortality. It was found that it did not impact the time until first hospitalization for HF exacerbation nor did it have an influence on mortality. Post hoc analysis of the TOPCAT study showed however that the hospitalization rate of patients randomized to spironolactone was reduced by 17%. The authors of the study go on to state that clinicians wanting to utilize spironolactone in the subpopulation of HF patients should be cognizant of the potential for hyperkalemia and increased serum creatinine, necessitating regular monitoring while on therapy [94]. Although sacubitril/valsartan is highly beneficial in the treatment of HFrEF patients, the PARAGON-HF trial revealed that it does not significantly lower the rate of total hospitalizations for heart failure and death from cardiovascular causes among these patients [95]. It has been hypothesized that the administration of short-term nitrate or inorganic nitrite may promote nitric oxide signaling, thus enhancing aerobic ability in patients with HFpEF. However, the administration of inhaled inorganic nitrite for 4 weeks, compared to placebo, also did not result in significant improvement in exercise capacity [96]. On the other hand, according to Nochioka et al., the treatment of HFpEF with statins reduces mortality [97]. Recent data reveal that anti-diabetic and anti-inflammatory drugs, anti-fibrotic and high-density lipoprotein-raising strategies, microRNases, mitochondrial-targeted anti-oxidants, and therapeutic options may be promising, although these warrant further investigations [98].

Interestingly, therapy with chlorthalidone has been found to prevent the occurrence of new-onset HFpEF in hypertensive patients [61]. Furthermore, in those subjects, ACEIs have shown promising results, namely lower blood pressure, decreased frequency of HF-related hospitalizations, improved exercise capacity, and diastolic function [56, 99].

The treatment of TTR amyloidosis is based on tafamidis, a drug that has been approved for use in patients with TTR polyneuropathy. In this condition, it has a significant impact on reducing symptoms and stabilizing TTR tetramers, and has been well-tolerated [100]. Findings from the ATTR-ACT study on ATTR cardiomyopathy show that tafamidis is associated with reduced mortality and cardiovascular-related hospitalizations. There are major benefits from the treatment if used in the early stage of the disease because of a reduction in the decline in functional capacity [101].

Emphasis is now being placed on the benefit of exercise therapy for patients with heart failure. This is in direct response to exercise intolerance being the primary symptom of patients with chronic HF and a major factor decreasing quality of life (QOL) in these patients [102]. Studies comparing endurance training in patients with HFpEF and HFrEF have shown a 19% improvement in peak VO_2_ in HFpEF after 12 weeks of exercise therapy. In contrast, no improvement was observed in the group with HFrEF [103]. The InterAtrial Shunt Device (IASD®), which reduces the elevated left atrial pressures, may also be promising [104, 105].

Due to the complex pathophysiology of HFpEF, multiple treatment strategies are still needed and will be required to target specific mechanisms of disease. As described in the Framingham Heart and the Cardiovascular Health Studies, the incidence of HFrEF has been declining (*p* = 0.0029), while the incidence of HFpEF is on the rise (*p* < 0.001). These trends were noticed from 1990 to 2009 [106]. It is necessary to discover the pathomechanisms responsible for this divergent trend. Until we are familiar with the pathways involved in this multifactorial disease, we can only recommend medications for our patients, which are known to work in other subtypes of HF. Needless to say, therefore, the treatment of comorbidities is of utmost importance. Recent data suggest that heart failure disease management programs may improve mortality, number of hospitalizations, self-care, and quality of life [107, 108]. However, it must be emphasized that there is currently no evidence-based therapy for HFpEF [109].

## Prognosis

Some sources report that both HF groups have similar outcomes, prognosis, and survival [8, 9, 110, 111]. On the contrary, the other studies point out that patients with HFpEF have a much better prognosis than patients with HFrEF [112, 113]. Somaratne et al. suggest that the survival rate of people with HFpEF is 50% higher compared to patients with HFrEF [114]. Although survival in HFrEF has significantly improved over the past decade, the prognosis of patients with HFpEF has not shown any notable change within the same time period despite the use of similar pharmacotherapy. The annual mortality of HFpEF patients in the USA is 8–12% [115]. In a major observational study, 5-year survival rate of HFpEF patients after hospitalization for HF was only 35–40%. Lack of evidence-based therapeutic strategies may play a pivotal role in curbing high rates of mortality and morbidity in HFpEF [8].

The identified prognostic factors in patients with HFpEF are as follows:

- Cystatin C (high serum level confers worse prognosis)
- B-type natriuretic peptide
- NT-proBNP
- Diabetes
- Growth factor 15 (GDF-15) [116–123]

Compared to patients with HFrEF, patients with HFpEF show lower levels of both B-type natriuretic peptide and NT-proBNP. However, in both cases, they are an important prognostic factor [123, 124]. Factors such as reduced LV compliance and remodeling of right ventricle (RV) also have prognostic significance, adversely affecting the prognosis [125]. Other factors that worsen prognosis are the coexistence of ischemic heart disease, diabetes mellitus, and chronic renal failure [22, 126].

## Summary

Heart failure with preserved ejection fraction (HFpEF) is defined by a left ventricular ejection fraction ≥ 50% in the presence of clinical signs and/or symptoms of heart failure, diastolic dysfunction, or structural abnormality of the left ventricle (LV). However, the system of classifying HF according to LVEF has been recently challenged. Symptoms classically associated with HF include dyspnea, paroxysmal nocturnal dyspnea, orthopnea, and fatigue. Natriuretic peptides are elevated.

The most common underlying causes of the disease are coronary artery disease, valvular heart disease, and hypertension, while the most common comorbidities in this population include obesity, diabetes, atrial fibrillation, metabolic syndrome, chronic obstructive pulmonary disease, sleep-disordered breathing, renal dysfunction, and anemia. Amyloidosis, specifically ATTR amyloidosis, is also an increasingly common cause of HFpEF and must be excluded in patients suspected of HF. While the pathophysiology of HFpEF is still being uncovered, the role of systemic low-grade inflammation and microvascular damage related to endothelial dysfunction, oxidative stress, and myocardial remodeling and fibrosis seem to be important components. As the percentage of HFpEF grows, relative to all cases of HF, it is a diagnosis, which clinicians need to be cognizant of.

Due to the fact that several pathophysiological processes may lead to dyspnea, the differential diagnosis is necessary to exclude the non-cardiac etiologies. Not all cases of HFpEF will present acutely. To screen for subclinical heart failure risk, the H_2_FPEF score and the HFpEF nomogram may be utilized. As there may be other diseases that mimic or share clinical characteristics, diagnostic algorithms are useful in making the diagnosis of HFpEF. The existence of different phenotypes of HFpEF becomes important when deciding which diagnostic strategies to employ.

Currently there is no proven pharmacotherapy specifically for HFpEF. Current pharmacotherapy includes angiotensin-converting enzyme inhibitors/aldosterone receptor blockers (ACE-inhibitors/ARBs), calcium channel blockers (CCBs), and beta-blockers. These medications are being used among HFpEF patients because of the high cardiovascular risk and concomitant diseases seen in this population. Treatment with spironolactone, however, seems to be promising. Finally, exercise therapy is being studied for its possible role in the treatment of these patients.

Due to a lack of evidence-based treatment strategies for HFpEF, the mortality and morbidity associated with the disease have remained high. The 5-year survival rate among patients with HFpEF is 35–40% after hospitalization. Further studies, especially with the use of machine learning, are warranted to investigate other underlying processes that lead to HFpEF as well as targeted pharmacotherapy for patients with HFpEF.
